# Severe asparaginase-associated hypertriglyceridemia in pediatric acute lymphoblastic leukemia: a single-center experience

**DOI:** 10.3389/fonc.2026.1845125

**Published:** 2026-06-23

**Authors:** Maha Barbar, Dana Kanaan, Zebin AlZebin, Ammar Al Hmood, Sima Kalaldeh, Lana Amer, Saja Al Zghoul, Dana Aroury

**Affiliations:** 1 Department of Pediatrics, King Hussein Cancer Center, Amman, Jordan; 2 Department of Pediatrics, School of Medicine, University of Jordan, Amman, Jordan

**Keywords:** acute lymphoblastic leukemia, asparaginase, children, hypertriglyceridemia, insulin therapy, Jordan, plasmapheresis

## Abstract

**Background:**

Asparaginase-associated hypertriglyceridemia (AAHTG) is a recognized metabolic complication of asparaginase therapy in pediatric acute lymphoblastic leukemia (ALL). However, data on its incidence, clinical presentation, and optimal management remain limited.

**Methods:**

We performed a retrospective analysis of 12 pediatric patients (≤18 years) with ALL treated at King Hussein Cancer Center (KHCC) between January 2021 and February 2026 who developed severe AAHTG, defined as triglyceride levels >1,000 mg/dL following asparaginase administration. Clinical characteristics, management strategies, and outcomes were systematically reviewed and analyzed.

**Results:**

Among 598 patients with pediatric ALL treated at KHCC, 12 developed severe AAHTG (incidence 2%). Most cases (83%) were asymptomatic and identified incidentally through observation of lipemic serum on routine laboratory testing; only two patients were symptomatic—one with acute pancreatitis and one with hyperviscosity syndrome. The median age was 9.5 years, with a male predominance (83%). Most patients had B-cell ALL (75%) and high-risk disease (66%). AAHTG occurred predominantly during the maintenance phase (66%), with a median onset of 12 days after the last asparaginase dose. The median peak triglyceride level was 2,645 mg/dL (range, 2,052–6,687 mg/dL). All patients received intravenous fluids, omega-3 fatty acids, and fibrates. Continuous insulin infusion was administered in 11 patients over a median of 3 days until triglyceride levels fell below 1,000 mg/dL. Plasmapheresis was utilized in three patients—two presenting with triglycerides exceeding 6,000 mg/dL and one with hyperviscosity syndrome—with all three achieving a reduction below 1,000 mg/dL after a single treatment session. Asparaginase was resumed per protocol in eight patients; recurrence occurred in one. Permanent discontinuation was required in four patients: two with symptomatic disease, one with refractory hypertriglyceridemia, and one who developed AAHTG after the final scheduled dose.

**Conclusion:**

Severe AAHTG is frequently asymptomatic and most often identified incidentally through lipemic serum; however, it carries a risk of serious complications. Early identification and prompt intervention were associated with favorable outcomes in this cohort. Resumption of asparaginase following an AAHTG episode appears feasible, provided triglyceride levels are confirmed below 1,000 mg/dL before each subsequent dose.

## Introduction

Acute lymphoblastic leukemia (ALL) is the most common malignancy of childhood. Treatment advances over recent decades have substantially improved outcomes, with contemporary protocols achieving 5-year survival rates exceeding 90% (1).

Asparaginase is a cornerstone of pediatric ALL therapy, exerting its antileukemic effect by catalyzing the hydrolysis of circulating asparagine to aspartate and ammonia, thereby exploiting the limited asparagine synthetase activity of leukemic lymphoblasts and depriving them of an amino acid essential for protein synthesis (2).

Despite its established benefit in improving survival, asparaginase is associated with multiple toxicities, including hypertriglyceridemia. The reported incidence during asparaginase-based regimens ranges from 10% to 50%, while severe hypertriglyceridemia (triglycerides >1,000 mg/dL) has been reported in up to 19% of patients (2, 3). Furthermore, the 2026 American Heart Association Scientific Statement on pediatric hypertriglyceridemia classifies triglyceride levels exceeding 2,000 mg/dL as very severe, a threshold associated with a substantially increased risk of acute pancreatitis (4).

The pathogenesis of asparaginase-induced hypertriglyceridemia is multifactorial, involving synergistic metabolic disturbances. Asparaginase suppresses lipoprotein lipase (LPL) activity, impairing clearance of triglyceride-rich lipoproteins and very-low-density lipoproteins (VLDLs), while simultaneously increasing hepatic VLDL synthesis and secretion (5).

Differences among asparaginase formulations may further influence the risk and severity of hypertriglyceridemia. Pegylated asparaginase (PEG-ASP), by virtue of its prolonged half-life and sustained enzymatic activity, may produce more persistent metabolic disturbances than native *Escherichia coli* or *Erwinia*-derived formulations (2).

Clinically, asparaginase-associated hypertriglyceridemia (AAHTG) ranges from incidental asymptomatic laboratory findings to potentially life-threatening complications, including acute pancreatitis, thrombosis, and hyperviscosity syndrome (6, 7).

In this case series, we describe 12 patients with pediatric ALL treated at King Hussein Cancer Center (KHCC) who developed severe AAHTG, detailing their clinical characteristics, management strategies, and outcomes. We also present a review of the published literature to further characterize the incidence, management, and prognosis of this complication.

## Methods

We conducted a retrospective review of all pediatric patients (≤18 years) with ALL treated at KHCC between January 2021 and February 2026 who developed severe or very severe AAHTG, defined as triglyceride levels exceeding 1,000 and 2,000 mg/dL, respectively, following asparaginase exposure, in accordance with the 2026 AHA Scientific Statement on pediatric hypertriglyceridemia (4). As routine post-asparaginase triglyceride monitoring is not incorporated into the KHCC ALL treatment protocol, AAHTG was identified either incidentally through lipemic serum detected during routine blood work, or through targeted testing prompted by clinical symptoms.

Demographic, clinical, and laboratory data were extracted from medical records and included leukemia immunophenotype, risk stratification, treatment phase at AAHTG onset, baseline triglyceride level (defined as the value documented at diagnosis prior to induction chemotherapy, where available), peak triglyceride levels, time from last asparaginase dose to AAHTG detection, clinical manifestations, therapeutic interventions, associated complications, and outcomes following asparaginase re-exposure. The study received approval from the KHCC Institutional Review Board.

A systematic literature search was also performed in PubMed, MEDLINE, and Google Scholar for studies published between January 1994 and February 2026. The search terms used included “asparaginase”, “hypertriglyceridemia”, “pediatric”, and “acute lymphoblastic leukemia”.

We included reports involving children (≤18 years) with severe hypertriglyceridemia (triglycerides >1,000 mg/dL) associated with asparaginase therapy. Studies involving adult patients, those lacking detailed patient-level data, or those not reporting triglyceride levels were excluded.

## Results

Of 598 patients with pediatric ALL treated at KHCC between January 2021 and February 2026 under the modified St. Jude Total XV protocol, 12 developed severe AAHTG following asparaginase administration, corresponding to an observed incidence of 2%. PEG-ASP was the sole formulation used.

The cohort comprised 10 male and 2 female patients, with a median age of 9.5 years (range, 2.5–18 years). B-cell ALL was the most common immunophenotype, observed in 9 of 12 patients (75%), and 8 patients (66%) were classified as having high-risk leukemia. AAHTG occurred most frequently during the maintenance phase (eight patients, 66%), while two patients developed it during relapse reinduction and two during initial induction. Six patients (50%) were overweight or obese, defined as body mass index (BMI) at or above the 85th percentile for age and sex at diagnosis. Baseline triglyceride levels, defined as measurements obtained prior to initiation of induction chemotherapy, were available for seven patients; of these, five had elevated levels (>150 mg/dL), with values ranging from 55 to 536 mg/dL (**Table 1**).

**Table 1:** Demographic and clinical characteristics of pediatric ALL patients with severe asparaginase-associated hypertriglyceridemia.

Case	Age (yr)	Gender	ALL immunophenotype	ALL risk	BMI percentile (age/sex)	Baseline TG (mg/dL)
1	5	M	B-ALL	LR	50th	N/A
2	2	F	B-ALL	HR	85th	N/A
3	10	M	T-ALL	HR	25th	N/A
4	8	M	T-ALL	HR	97th	N/A
5	15	M	T-ALL	HR	97th	278
6	13	M	B-ALL	HR	95th	102
7	16	M	B-ALL	HR	97th	536
8	10	F	B-ALL	LR	97th	55
9	3	M	B-ALL	HR	10th	519
10	4	M	B-ALL	SR	10th	126
11	9	M	B-ALL	SR	75th	200
12	18	M	B-ALL	HR	66th	244

Only two patients exhibited clinical symptoms: one presented with concurrent hyperglycemia, metabolic acidosis, and acute pancreatitis, while the other developed headache and blurred vision consistent with hyperviscosity syndrome (**Table 2**).

**Table 2:** Clinical presentation, management, and outcomes of severe asparaginase-associated hypertriglyceridemia.

Case	ALL treatment phase of AAHTG	Presentation	Peak TG level (mg/dL)	Onset interval post-asparaginase (days)	Management		Time to decrease TG <1000 (mg/dL) (days)	Time to normalize TG (<150 mg/dL) (days)
					Insulin (duration, days)	Plasmapheresis (no. of sessions)		
1	Maintenance phase (week 4)	Lipemic serum	2052	14	2 days		2	5
2	Maintenance phase (week 4)	Lipemic serum	2696	14	5 days		5	8
3	Induction phase (week 1)	Lipemic serum	6554	10	1 day	1 session	1	3
4	Re-induction phase (week 3)	Hyperglycemia, acidosis, acute pancreatitis	2553	14	2 days		2	14
5	Maintenance phase (week 4)	Persistent transaminitis	3124	21	3 days		4	90
6	Induction phase (week 5)	Lipemic serum	6687	10	1 day	1 session	1	5
7	Re-induction phase (week 4)	Lipemic serum	2229	14	9 days		9	180
8	Maintenance phase (week 7)	Metabolic syndrome evaluation	2594	5	1 day		1	4
9	Re-induction phase (week 1)	Lipemic serum	2140	9	3 days		3	90
10	Maintenance phase (week 20)	Lipemic serum	2300	14	4 days		3	60
11	Maintenance phase (week 20)	Lipemic serum	4115	7	3 days		3	8
12	Maintenance phase (week 14)	Pseudohyponatremia, headache, blurred vision	3900	7		one session	1	56

Peak triglyceride levels ranged from 2,052 to 6,687 mg/dL (median, 2,645 mg/dL); all 12 patients met the threshold for very severe hypertriglyceridemia (triglycerides >2,000 mg/dL), including 2 with levels exceeding 6,000 mg/dL. The median interval from the last asparaginase dose to AAHTG detection was 12 days (range, 5–21).

Given that all patients met criteria for very severe hypertriglyceridemia—including those who were asymptomatic—all were admitted to the pediatric intensive care unit (PICU) for close monitoring and prompt triglyceride reduction. PICU-level care was further warranted by the risk of serious complications, including pancreatitis, pseudohyponatremia, and thrombosis, and by the requirement for continuous insulin infusion with intensive glucose surveillance.

Management comprised intravenous hydration, dietary fat restriction, and combination lipid-lowering therapy including omega-3 fatty acid supplementation. Continuous insulin infusion was initiated in 11 patients at 0.01 IU/kg/h with concurrent 10% dextrose infusion. Serum glucose was monitored every 2 h and hourly if hypoglycemia was suspected. Although no patient developed symptomatic hypoglycemia, asymptomatic hypoglycemia was documented in 80% of cases.

Emergent plasmapheresis was performed in the patient presenting with hyperviscosity symptoms. Two additional patients with triglyceride levels exceeding 6,000 mg/dL underwent plasmapheresis within 24 h of presentation, after which insulin infusion was discontinued. The remaining patients continued insulin infusion until triglyceride levels fell below 1,000 mg/dL, with a median duration of 3 days (range, 1–9).

All three patients underwent plasmapheresis without complications and achieved triglyceride reduction below 1,000 mg/dL after a single session. Complete normalization of triglyceride levels (<150 mg/dL) required a longer duration of lipid-lowering therapy, omega-3 supplementation, and dietary fat restriction, ranging from 3 days to 6 months (median, 8 days). No patient developed new acute complications—including pancreatitis or thrombosis—attributable to hypertriglyceridemia during management, apart from the one patient who had presented with pancreatitis at onset.

Eight patients were re-exposed to asparaginase per protocol following AAHTG resolution; recurrence occurred in one (12.5%), leading to omission of all remaining doses.

Four patients did not receive further asparaginase after their AAHTG episode. Asparaginase was discontinued in the two symptomatic patients (one with acute pancreatitis and one with hyperviscosity syndrome) and in a third patient with persistent severe hypertriglyceridemia refractory to all interventions. The fourth patient developed AAHTG after the final scheduled asparaginase dose per protocol and required no further exposure.

Over a median follow-up of 16 months, one patient developed asparaginase-induced pancreatitis without concurrent hypertriglyceridemia during treatment for relapsed disease. No cases of avascular necrosis or thrombosis were observed.

## Discussion

AAHTG is an established metabolic complication in children with ALL. The reported incidence of hypertriglyceridemia ranges from 10% to 67% (7), while severe hypertriglyceridemia (triglycerides >1,000 mg/dL) has been reported in approximately 4%–19% of patients (2).

Among 598 patients with pediatric ALL treated at KHCC with PEG-ASP per the modified St. Jude Total XV protocol, 12 cases of severe AAHTG were identified, corresponding to an observed incidence of 2%. However, as triglyceride monitoring after asparaginase administration is not routinely incorporated into the leukemia treatment protocol at KHCC, the true incidence is likely higher as asymptomatic cases without incidentally detected lipemic serum may have remained unrecognized.

A review of the literature published over the past three decades (1994–February 2026) identified 14 publications describing 28 cases of AAHTG in pediatric patients with ALL. The addition of 12 cases from the present cohort substantially expands the limited published data on this complication. Male patients predominated in our series (83%), whereas previously reported cohorts demonstrated a female predominance (61%) (**Table 3**).

**Table 3:** Previously reported cases of AAHTG in pediatric ALL.

Reference/author	Publication year	Number of cases reported	Age (yr)	Gender (F:M)	Type of asparaginase formulation	Time interval from asparaginase dose to TG peak (days)	Presentation	TG peak (mg/dL)	Treatment	
									Medical	Plasmapheresis
8 - Sie Chong Doris Lau	2021	2	12, 13	M	L-ASP	1st: 20 days	1st: Asymptomatic	1st 1327	SMOF lipid, low-fat diet	No
						2nd: 30 days	2nd: Transaminitis	2nd 1371		
9 - Ashley Hinson	2014	2	11, 3	F	PEG-ASP	1st: 30 days	Pseudohyponatremia	1st 3636	Fibrate therapy, low-fat diet	No
						2nd: 21 days		2nd 3237		
10- Harsha Prasad	2011	5	Median 8 (range 3–16)	2M: 3F	PEG-ASP	Median 21 days	Lipemic serum in all patients	Median 2565 (1,800–3,700)	Statins in 5	No
							Pseudohyponatremia (n=4); lipemic serum (n=1); incidental finding (n=3)		Heparin infusion in 1	
11 - Joanna Zawitkowska	2019	2	5,10	M F	PEG-ASP	1st: 28 days	Pseudohyponatremia	1st 9300	Low-fat diet	yes
						2nd: 14 days		2nd 8000	fibrate	
12 - Goutham KJ	2022	1	2	M	PEG-ASP	28 days	Lipemic serum	3324	Low-fat diet	No
13 - Morand	2016	1	6	F	L-ASP	21 days	Lipemic serumPseudohyponatremia	1250	Low-fat diet, fibrates	Yes
14 - Solano-Paez	2011	1	10	F	L-ASP	14 days	Neurological symptoms	9250	Fibrate, Omega-3, Low-fat diet	Yes
15 - Vita Ridola	2006	1	10	M	L-ASP	14 days	Lipemic serum	4000	Low-fat diet	Yes
16 - Peter G. Steinherz	1994	1	10	F	L-ASP	21 days	Lipemic serum	20000	Heparin infusion	No
17 - Fani Athanassiadou	2004	1	10	F	L-ASP	21 days	Asymptomatic (incidental finding)	1817	Fibrate, omega-3	No
18 - David H. Abramson	1995	1	10	F	L-ASP	10 days	Lipemic serum	2700	Omega-3, fibrate, Low-fat diet	Yes
19 - Gabriele Kropshofer	2003	1	16	F	L-ASP	21 days	Lipemic serum	8510	Niacin	Yes
20 - Sandeep Jain	2009	1	17	F	L-ASP	28 days	Lipemic serum	5250	Lipid-lowering agents	No
21 - Amr Elgehiny	2025	8	Median 12(range 2–14)	5M: 3F	CAL-ASP (n=7); PEG-ASP (n=1)	Median 17 (3–69)	Pseudohyponatremia in 4 patients; Lipemic serumLipemic serum (n=1); incidental finding (n=3)	Median 2154 (range 1082–3965)	Fibrate, omega-3, statins; insulin (n=2); L-carnitine (n=7)	No

AAHTG, asparaginase-associated hypertriglyceridemia; L-ASP, native Escherichia coli L-asparaginase; PEG-ASP, pegylated asparaginase; CAL-ASP, calaspargase pegol; TG, triglycerides; F, female; M, male; SMOF, soybean oil/MCT/olive oil/fish oil lipid emulsion.

Most patients in our cohort (83%) were asymptomatic, with severe hypertriglyceridemia detected incidentally. In eight patients, lipemic serum was noted during routine laboratory investigations, whereas in the remaining two asymptomatic patients, triglyceride levels were obtained during evaluation for metabolic syndrome and persistent transaminitis, respectively. These findings are consistent with previous reports showing that even markedly elevated triglyceride levels may remain clinically silent. In pooled analyses of published cases, 64% of patients were asymptomatic, and among these, 72% were identified primarily through the detection of lipemic serum—a pattern similar to that observed in our cohort. Symptomatic severe hypertriglyceridemia occurred in only two patients (17%): one presented with hyperglycemia, metabolic acidosis, and acute pancreatitis, while the other developed hyperviscosity symptoms manifested by headache and blurred vision.

Pseudohyponatremia has been reported in 35% of published cases (**Table 3**). In contrast, only one patient in our cohort developed pseudohyponatremia; this patient presented with hyperviscosity symptoms and a triglyceride level of 3,900 mg/dL.

In our cohort, severe AAHTG occurred most commonly during the maintenance phase (66%) of the modified St. Jude Total XV protocol, likely reflecting the scheduling of PEG-ASP doses within this phase of our institutional protocol. Among previously published cases where the treatment phase was specified, AAHTG appeared to occur more frequently during induction. (8–13) However, direct comparison is limited by the heterogeneity of treatment protocols across reported cohorts. AAHTG has been reported with all available asparaginase formulations (**Table 3**), including native *E. coli* L-asparaginase (L-ASP) (8, 13–20) and PEG-ASP (9–12, 21). In our cohort, PEG-ASP was the sole formulation used, and the median interval from the last asparaginase dose to the onset of severe AAHTG was 12 days (range, 5–21 days). This interval was shorter than the median of 21 days reported in previously published cases involving both L-ASP and PEG-ASP. Specifically, reported onset ranges were 10–30 days for L-ASP and 24–30 days for PEG-ASP (**Table 3**).

AAHTG has recently been documented with calaspargase pegol (CAL-ASP). Seven severe AAHTG events following CAL-ASP administration were reported by the MD Anderson Cancer Center (21), with a median onset of 17 days (range, 3–69 days). The relatively extended and wider onset window observed with CAL-ASP likely reflects its prolonged half-life and more sustained enzymatic activity compared with other asparaginase formulations.

In our cohort, peak triglyceride levels ranged from 2,052 to 6,687 mg/dL, with a median of 2,645 mg/dL compared with a range of 1,250 to 20,000 mg/dL and a median of 3,324 mg/dL in previously published cases. As noted, triglyceride levels exceeding 2,000 mg/dL are classified as very severe per the 2026 AHA Scientific Statement, and all patients in this cohort met that threshold (4).

Pre-existing metabolic risk factors may have contributed to AAHTG severity in our cohort. Baseline triglyceride levels were available for 7 of the 12 patients; among them, 5 had elevated triglyceride levels (55–536 mg/dL; normal <150 mg/dL) prior to the first asparaginase dose. In addition, six patients (50%) had a BMI at or above the 85th percentile at diagnosis. Concurrent corticosteroid therapy represents another important confounding factor, as all patients received steroids during both induction and maintenance phases. Consequently, the individual contribution of steroids cannot be clearly distinguished from that of asparaginase. Therefore, severe and very severe AAHTG in this context is likely multifactorial, with asparaginase serving as the principal precipitating factor in patients with underlying metabolic susceptibility.

The primary goal of management in severe AAHTG is to reduce the risk of acute pancreatitis. At triglyceride levels exceeding 885 mg/dL, LPL becomes unable to clear chylomicrons effectively, leading to their accumulation and a progressive increase in pancreatitis risk. Levels exceeding 2,000 mg/dL are classified as very severe and are associated with a substantial rise in both the relative and absolute risk of acute pancreatitis (4). Management of severe AAHTG ranges from conservative to more aggressive interventions, depending on the severity of symptoms and associated complications. Conservative approaches include dietary modification, oral lipid-lowering agents such as omega-3 fatty acids, fibrates, and statins. In patients with very severe hypertriglyceridemia, particularly those who are symptomatic or at high risk for complications, more intensive therapies including insulin infusion, heparin infusion, and therapeutic plasma exchange (plasmapheresis) have been employed (22) (**Table 3**).

Insulin reduces triglyceride levels through multiple mechanisms, including stimulation of LPL activity, suppression of peripheral lipolysis, and reduction of hepatic triglyceride synthesis, collectively promoting rapid clearance of circulating triglycerides (23, 24). Given the combined pancreatitis risk associated with very severe hypertriglyceridemia and asparaginase therapy, rapid triglyceride reduction was prioritized in all patients. Eleven patients received continuous insulin infusion at 0.01 IU/kg/h in combination with 10% dextrose. Insulin therapy was continued until triglyceride levels decreased to below 1,000 mg/dL. Serum glucose was monitored hourly, with insulin infusion rates and dextrose concentrations adjusted accordingly. The median duration of insulin infusion was 3 days (range, 1–9 days). Although no patient developed symptomatic hypoglycemia, asymptomatic hypoglycemia was documented in 80% of cases.

Evidence supporting the use of therapeutic plasma exchange (plasmapheresis) in pediatric AAHTG is limited and primarily derived from case reports and small case series (13–15, 19, 24). The decision to pursue plasmapheresis is influenced by several factors, including the severity of hypertriglyceridemia, the presence of complications such as pancreatitis or hyperviscosity symptoms, and the patient’s age and body weight. In addition, the feasibility and safety of plasmapheresis may be challenging in pediatric oncology patients because of concurrent coagulopathy or cytopenias. Plasmapheresis was performed in three patients in our cohort (25%): two because of markedly elevated triglyceride levels exceeding 6,000 mg/dL, and one due to hyperviscosity symptoms. Although it may have been indicated, plasmapheresis was not feasible in the patient who presented with metabolic acidosis and pancreatitis because of clinical instability. All three patients who underwent plasmapheresis were male individuals older than 10 years and required only a single session, each achieving rapid triglyceride reduction below 1,000 mg/dL.

Among previously reported cases (**Table 3**), plasmapheresis was performed in seven patients (25%), with a median age of 10 years and a median peak triglyceride level of 4,020 mg/dL. The indications included pancreatitis in two patients, (13, 15) neurological symptoms in one, (14) deteriorating clinical status in one, (19) and extreme triglyceride elevation in the remainder.

SMOF lipid (soybean oil, MCT, olive oil, and fish oil) and omega-3 fatty acids have also been employed in AAHTG cases (8, 14, 17, 18, 21). Their proposed mechanism involves reduction of hepatic VLDL synthesis and enhancement of triglyceride clearance. All patients were treated with omega-3 fatty acids in combination with fibrates, statins, and dietary fat restriction until triglyceride normalization (<150 mg/dL), achieved after a median of 8 days (range, 3 days to 6 months).

Heparin infusion has also been reported as an alternative strategy for AAHTG management (10, 16). Heparin transiently releases endothelial LPL into the circulation, enhancing hydrolysis of triglyceride-rich lipoproteins; however, its effect is short-lived and it carries a significant bleeding risk in oncology patients with coagulopathy or thrombocytopenia.

Given the potential for AAHTG recurrence with subsequent asparaginase doses, KHCC implemented routine triglyceride monitoring before each subsequent administration. Eight of 12 patients were re-exposed to asparaginase per protocol once triglyceride levels had fallen below 1,000 mg/dL. Recurrence occurred in only one patient (12.5%), prompting omission of the single remaining scheduled dose.

Four patients did not receive further asparaginase following their AAHTG episode. Treatment was discontinued in two symptomatic patients—one who developed acute pancreatitis and another who presented with hyperviscosity symptoms. In a third patient, asparaginase was omitted due to refractory severe hypertriglyceridemia.

By contrast, the MD Anderson Cancer Center reported 18 episodes of AAHTG among eight patients, indicating recurrence after re-administration of asparaginase in four patients; notably, one patient experienced six separate AAHTG events. Despite these recurrences, asparaginase was not permanently omitted in any patient and was continued per protocol once triglyceride levels decreased below 1,000 mg/dL.

Asymptomatic AAHTG should not preclude continuation of asparaginase therapy, provided triglyceride levels are confirmed below 1,000 mg/dL before each subsequent dose and institutional criteria for dose omission or permanent discontinuation are defined prospectively.

### Limitations

This study has several limitations. First, the retrospective single-center design limits generalizability and carries an inherent risk of incomplete data capture. Second, as triglyceride monitoring after asparaginase is not routine at KHCC, AAHTG was captured only when lipemic serum was incidentally detected; cases where triglycerides rose and fell between blood draws would have been missed, and the observed incidence of 2% should be regarded as a minimum estimate. Third, baseline triglyceride data were available in only 7 of 12 patients, limiting assessment of pre-existing dyslipidemia as a risk factor. Fourth, the small cohort size precludes multivariable analysis of predictors for symptom development, plasmapheresis requirement, or recurrence on re-exposure. Fifth, the absence of a comparator group means no conclusions can be drawn about the relative efficacy of individual interventions. Prospective multicenter registries are needed to establish true incidence, identify risk predictors, and define evidence-based monitoring and rechallenge thresholds for AAHTG in children receiving asparaginase-based therapy.

## Conclusion

Severe and very severe AAHTG in pediatric ALL is frequently asymptomatic and most often identified incidentally through lipemic serum. Pseudohyponatremia was reported in 35% of previously published cases and was documented in one patient in our cohort; its presence should serve as a clinical prompt for triglyceride evaluation following asparaginase administration.

In this retrospective single-center series, a management approach combining insulin infusion, lipid-lowering agents, and dietary fat restriction was associated with triglyceride normalization in all treated patients; however, the uncontrolled study design precludes conclusions regarding the efficacy of individual interventions. Plasmapheresis was reserved for hyperviscosity symptoms or extremely severe hypertriglyceridemia, achieving rapid triglyceride reduction in all cases where performed.

Asymptomatic AAHTG should not preclude asparaginase continuation, provided triglyceride levels are confirmed below 1,000 mg/dL before each subsequent dose. This observation is based on a small cohort and warrants confirmation in larger prospective studies. Multicenter registries are needed to establish standardized monitoring thresholds and rechallenge criteria for AAHTG in children receiving asparaginase-based therapy.

## Data Availability

The raw data supporting the conclusions of this article will be made available by the authors, without undue reservation.
